# Population-based screening for colorectal cancer in Wuhan, China

**DOI:** 10.3389/fonc.2024.1284975

**Published:** 2024-02-29

**Authors:** Song Liu, Yifan Wang, Yuying Wang, Chaofan Duan, Fan Liu, Heng Zhang, Xia Tian, Xiangwu Ding, Manling Zhang, Dan Cao, Yi Liu, Ruijingfang Jiang, Duan Zhuo, Jiaxi Peng, Shida Zhu, Lijian Zhao, Jian Wang, Li Wei, Zhaohong Shi

**Affiliations:** ^1^ Department of Gastroenterology, Wuhan No. 1 Hospital, Wuhan Hospital of Traditional Chinese and Western Medicine, Wuhan, Hubei, China; ^2^ Wuhan No. 1 Hospital, Wuhan Hospital of Traditional Chinese and Western Medicine, Tongji Medical College, Huazhong University of Science and Technology, Wuhan, Hubei, China; ^3^ BGI Genomics Co., Ltd., Shenzhen, China; ^4^ Department of Gastroenterology, Wuhan Central Hospital, Wuhan, Hubei, China; ^5^ Department of Gastroenterology, The Third Hospital of Wuhan (Tongren Hospital of Wuhan University), Wuhan, Hubei, China; ^6^ Department of Gastroenterology, The Fourth Hospital of Wuhan, Wuhan, Hubei, China

**Keywords:** colorectal cancer screening, population-based study, fecal DNA based test, colonoscopy, risk factors

## Abstract

Fecal DNA test has emerged as a non-invasive alternative for colorectal cancer (CRC) screening in average-risk population. However, there is currently insufficient evidence in China to demonstrate the effectiveness of population-based CRC screening using fecal DNA based test. Here, a large-scale real-world study for CRC screening was implemented in Wuhan, Hubei province, China. A total of 98,683 subjects aged between 45 and 60 years were screened by a fecal DNA test (ColoTect^®^) which detected methylation status of *SDC2*, *ADHFE1*, and *PPP2R5C*. Participants who tested positive were advised to receive diagnostic colonoscopy. 4449 (4.5%) subjects tested positive for fecal DNA test, and 3200 (71.9%) underwent colonoscopy. Among these, 2347 (73.3%) had abnormal colonoscopy findings, of which 1330 (56.7%) subjects received pathological diagnosis. Detection rates for CRC and advanced precancerous lesions were 1.3% and 2.3%, respectively. Detection rates for nonadvanced adenomas and polyps were 14.0% and 21.6%, respectively. 28.0% of all colonoscopies showed colorectal neoplasm but lack pathological diagnosis. 6.1% showed other abnormalities such as enteritis. In conclusion, preliminary real-world evidence suggested that fecal DNA tests had promising diagnostic yield in population-based CRC screening.

**Clinical trial registration:**

https://www.chictr.org.cn/showproj.html?proj=192838, identifier ChiCTR2300070520.

## 1 Introduction

Colorectal cancer (CRC) is the third common cancer and the second leading cause of cancer-related death worldwide, with 1.9 million new cases and 935,000 deaths in 2020 (1). The incidence and mortality of CRC in China have been increasing in China in the past decade, representing 28.8% of new cancer cases and 30.6% of CRC-related deaths in the world in 2020 (1). In addition, the survival rate is closely related to the stage of cancer at diagnosis (2). The 5-year relative survival rate for the distant stage CRC is less than 10%, whereas that for the localized stage disease is higher than 90% (3). However, the detection rate of early-stage CRC in China remains less than 10%, which is far behind the average (4, 5). CRC, therefore, posing a massive challenge to the public health system in China. It is therefore of critical significance to implement efficient CRC screening strategies to help improve diagnosis and reduce the disease burden.

Notably, currently available CRC screening methods have certain limitations such as cost, lack of accessibility, and low screening compliance. Colonoscopy, the gold standard for CRC screening, is a costly and invasive procedure that requires experienced endoscopist. Due to the scarcity of colonoscopy resources, large-scale population screening by colonoscopy is impractical in China (6). Although non-invasive techniques (such as FIT) have a high compliance rate in the population, the sensitivity for detection of early-stage CRC and precancerous lesions were reported to be unsatisfactory (2) and subsequent compliance for colonoscopy in people who screened positive for FIT remains low.

To overcome these disadvantages of traditional CRC screening approaches, alternative methods have been developed for CRC screening. One example is the multi-target stool DNA test, approved by the FDA in 2014 in the United States, which provides higher sensitivity for detecting CRC and advanced precanserous lesions (APLs; 7–9). To date, authoritative guidelines have recommended fecal DNA testing followed by diagnostic colonoscopy for CRC screening of average risk populations in several countries (10, 11).

Here in this study, we conducted a large-scale, multi-center population-based CRC screening trial utilizing a novel fecal DNA-based test followed by diagnostic colonoscopy in Wuhan, Hubei Province, China. Meanwhile, to help elucidate the complex pathogenesis of colorectal neoplasms, we aimed to collect and integrate additional clinical information such as personal disease history and presence of relevant symptoms (12). The purpose was to develop a practical risk-adapted CRC screening strategy, and to provide a basis for formulating effective CRC screening strategies in the future.

## 2 Methods

### 2.1 Study design and population

A community-based CRC screening program was implemented in Wuhan city, Hubei Province, China, from November 2021 to March 2022. This program was organized by Wuhan Municipal Health Commission in Hubei Province and jointly implemented by local hospitals and community health centers. Residents aged 45-60 living in Wuhan and without history of CRC were eligible to participate in this program.

**Figure 1:**
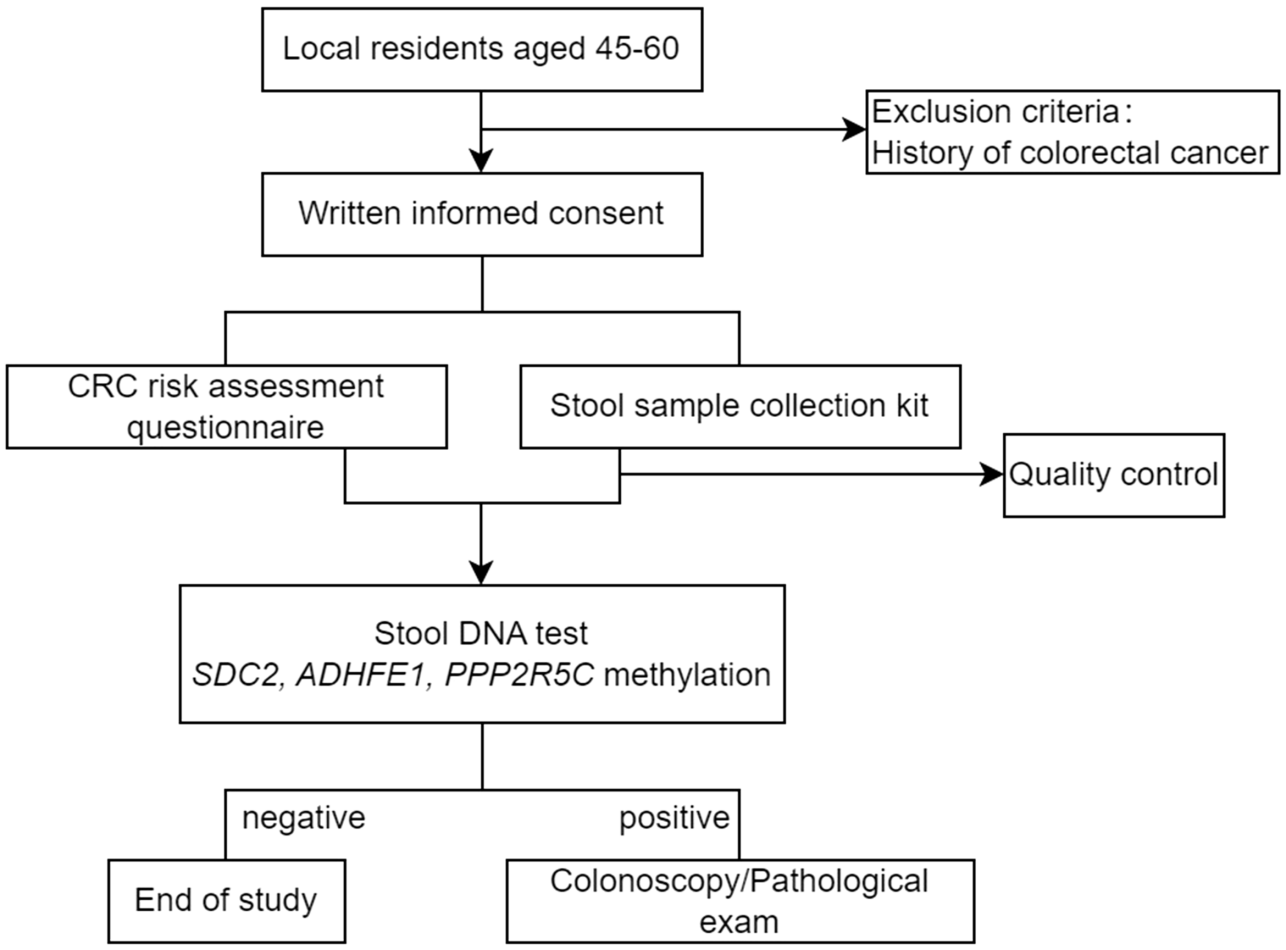
Study design of colorectal cancer screening for Wuhan permanent residents.

Briefly, people who were willing to participate in the CRC screening program first made an appointment and provided informed consent through a mobile custom app designed for the screening program. Participants were required to then completed an online CRC risk assessment questionnaire, before they received the stool sample collection kits (BGI Genomics Co., Ltd, Shenzhen, China). Self-collected stool samples were returned to community health centers and sent to the central lab where multi-gene fecal DNA tests (ColoTect^®^, BGI Genomics Co., Ltd, Shenzhen, China) were performed, which detect methylation status of *SDC2*, *ADHFE1*, and *PPP2R5C* in exfoliated intestinal cells. ColoTect^®^ achieved a sensitivity of 88% for detecting CRC and a sensitivity of 46% for detecting advanced precancerous lesions, at a specificity of 91.8% in a retrospective, case-control study (13). Participants who tested positive were advised to receive diagnostic colonoscopy at one of the 17 local hospitals. The flow chart of study population recruitment is shown in **Figure 1**.

### 2.2 Outcomes and quality control

Those with colorectal neoplasm during colonoscopy were considered as positive results, including colorectal cancers, advanced precancerous lesions, and non-advanced neoplastic findings (adenomas and polyps). In this study, advanced precancerous lesions refer to advanced adenomas, specifically serrated adenomas, villous adenoma, adenoma with size ≥ 1 cm, and high-grade intraepithelial neoplasia.

During this study, physicians of community health center were trained to collect DNA methylation test samples and later advise participants who tested positive to receive diagnostic colonoscopy under standard protocol. Colonoscopy and subsequent pathological diagnosis were performed by experienced endoscopists and pathologists, respectively. Diagnoses were provided according to current clinical guidelines.

### 2.3 Ethics

This study was approved by the Medical Ethics Committee of Wuhan Hospital of Integrated Traditional Chinese and Western Medicine. The local ethics committee of Wuhan Municipal Health Bureau reviewed and approved the research involving human participants. All investigations and methods used were implemented in accordance with the Helsinki Declaration. All patient/participant provided written informed consent to participate in the study.

### 2.4 Data collection

Before they received the stool sample collection kits, all participants were asked to provide basic information, including age, sex, height, body weight, and to answer questions for CRC risk stratification, including personal history of severe mental trauma within the past 20 years (*e.g.*, the death of spouse or first-degree relatives, divorce, unemployment), personal history of chronic constipation (defined as infrequent bowel movements or difficult passage of stools that persists for at least 2 months every year within pervious 2 years), personal history of intestinal polyps, chronic cholecystitis or cholecystectomy, chronic diarrhea (defined as loose/watery stools that lasts for 1 week or longer every time and for more than 3 months within previous 2 years), family history of CRC in a first-degree relative, chronic appendicitis or appendectomy, personal history of any cancer and bloody mucus in stools. As previously reported, participants who met any of the following criteria were considered high-risk groups for colorectal cancer: ① positive fecal occult blood; ② first-degree relatives with a history of colorectal cancer; ③ history of intestinal adenoma; ④ history of any cancer; ⑤ met any 2 of the following 6 criteria: chronic diarrhea, chronic constipation, mucus and bloody stools, chronic appendicitis or appendectomy history, chronic cholecystitis or cholecystectomy history, and long-term mental depression. Participants with a test score >=5 on the DNA methylation test were regarded to have a higher risk for CRC and advanced precancerous lesions and referred to designated hospitals for diagnostic colonoscopy examination. Biopsies were performed on lesions found to provide the pathological diagnosis.

### 2.5 Statistical analysis

EXCEL software was used to input and organize the data, and IBM SPSS Statistics 26 software was used to carry out statistical calculation, χ^2^ test, and logistic multivariate analysis on the results. P-values were two-sided and values below 0.05 were considered as statistically significant.

The following metrics were defined: (1) The positive rate (high risk rate) of screening = number of cases assessed as positive in fecal DNA test/total number of cases participating in the screening program × 100%; (2) The participation rate of colonoscopy = number of participants who received diagnostic colonoscopy/number of participants screened positive × 100%; (3) The rate of pathological diagnosis = number of cases that completed pathological examination/number of participants who had positive (neoplastic) findings in diagnostic colonoscopy × 100%. (4) Positive predictive value = number of participants with positive findings/number of participants who received diagnostic colonoscopy × 100%. Here, for PPV calculation, a “positive” result included findings of cancer or APLs, and a “negative” result included nonadvanced adenoma, polyps, enteritis, and normal findings in colonoscopy (14, 15).

## 3 Results

### 3.1 Characteristics of the study population

**Figure 2:**
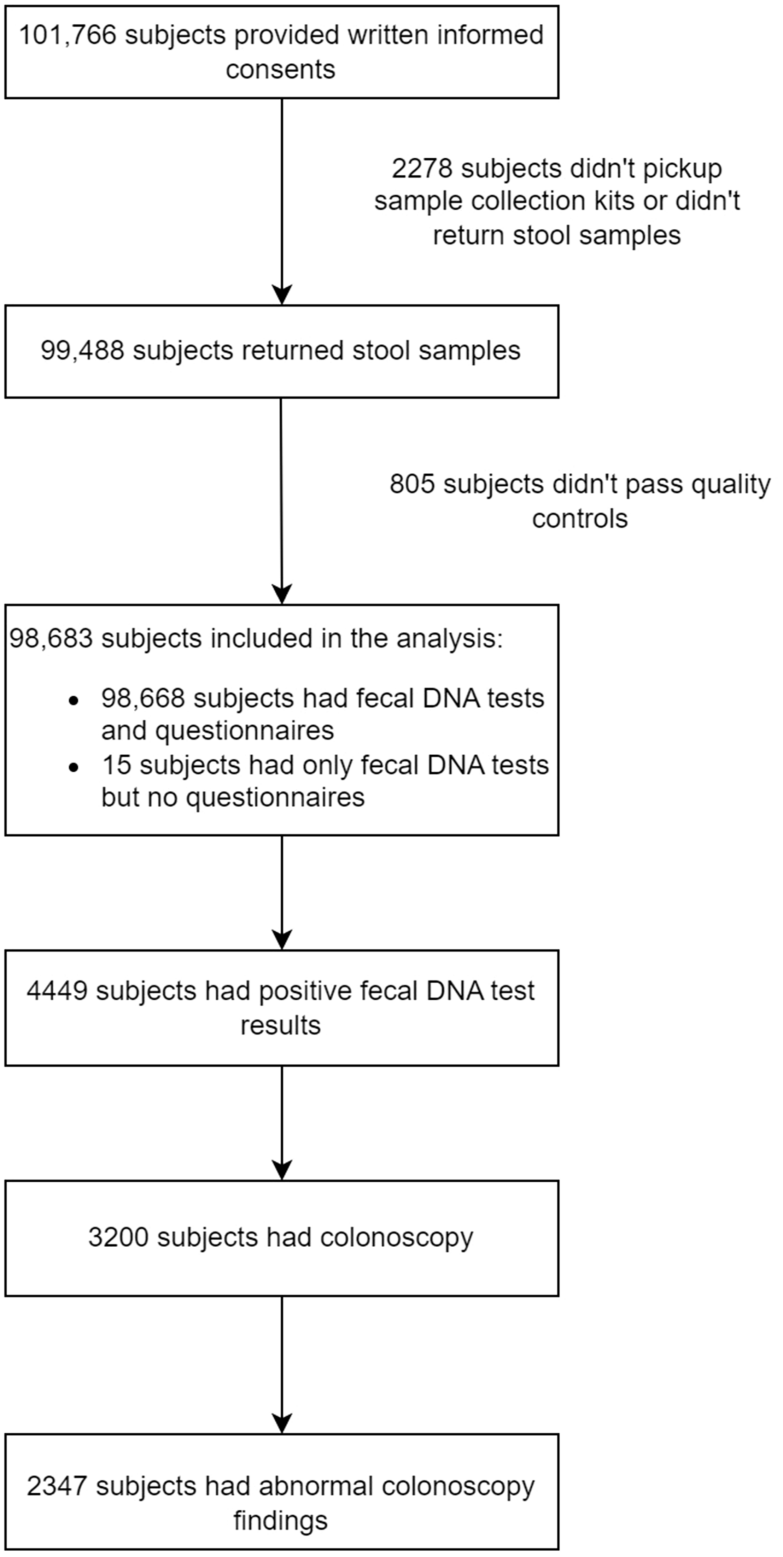
Flow diagram indicating the number of participants and summarizing the screening results as well as findings in colonoscopy.

A total of 101,766 subjects were enrolled, of whom 99,488 subjects returned stool specimens. Of 98,683 subjects provided qualified stool samples and received a result of fecal DNA test, 98,668 completed CRC risk assessment questionnaires. The median age of the study population was 52 years old. 4,434 (4.48%) subjects tested positive for fecal DNA test, and 3,200 (adherence rate = 72.2%) underwent colonoscopy. Among these, 2347 (73.3%) had abnormal colonoscopy findings, of which 1330 (56.7%) subjects received pathological diagnosis (**Figure 2**).

The baseline information of the 98,668 subjects was shown in **Table 1**. Overall, 61,331 (62.0%) participants were female and 37,644 (38.0%) participants were male, indicating that female in Wuhan were more likely to participate in a CRC screening program. The established risk factor of CRC was not observed in the majority of participants.

**Table 1:** Characteristics of the study population.

Indicator (#)		N (%)
Age		
	[45-50]	27682 (28.06)
	[50-53]	21840 (22.13)
	[53-57]	25189 (25.53)
	[57-60]	23957 (24.28)
Sex		
	female	61142 (61.97)
	male	37526 (38.03)
Questionnaire		
	low risk	78480 (79.54)
	High risk	20188 (20.46)
History of chronic constipation		
	no	83664 (84.79)
	yes	15004 (15.21)
Severe mental trauma within 20 years		
	no	90553 (91.76)
	yes	8135 (8.24)
History of polyps		
	no	92776 (94.03)
	yes	5892 (5.97)
History of chronic cholecystitis or cholecystectomy		
	no	93746 (95.01)
	yes	4922 (4.99)
History of chronic diarrhea		
	no	91745 (92.98)
	yes	6923 (7.02)
History of chronic appendicitis or appendectomy		
	no	93147 (94.40)
	yes	5521 (5.60)
History of cancer		
	no	96793 (98.10)
	yes	1875 (1.90)
Mucus/blood in stool		
	no	89840 (91.05)
	yes	8828 (8.95)
History of CRC in first-degree relatives		
	no	71996 (72.97)
	yes	4547 (4.61)
	NA	22125 (22.42)

NA, Not Applicable.

### 3.2 Diagnostic yield

Among 2347 participants who showed positive findings on colonoscopy, 1330 participants (56.7%) subsequently received pathological diagnosis; the rest did not receive pathological diagnosis because biopsy was deemed unnecessary by physicians or biopsy sample being too small for pathological examination or loss of follow-up. Overall, 1328 subjects (99.85%) had abnormal results in pathological examination. As shown in **Table 2**, out of 3200 colonoscopies performed, CRCs were found in 41 subjects (1.28%). Advanced precancerous lesions (APLs, defined as adenoma measuring ≥ 1cm in diameter, or with villous histology, or high-grade dysplasia, or serrated adenoma/polyp) were confirmed in 75 subjects (2.34%). Therefore, the positive predictive values (PPV) of the Colotect test was 1.28% for CRC, 2.34% for APLs, or 3.63% (116/3200) for advanced neoplasm (CRCs and APLs). Non-advanced adenomas were identified in 448 subjects (14.00% of all colonoscopies performed). Non-adenomatous polyps were found in 691 subjects (21.59%). Besides, 73 subjects showed other gastrointestinal lesions, such as enteritis (6.09%). Furthermore, 895 (27.97%) colonoscopy examinations showed colorectal neoplasm but lack pathological diagnosis. Meanwhile, 122 (3.81%) subjects showed other abnormal findings on colonoscopy but had no pathological diagnosis.

**Table 2:** Number of colonic lesions detected by colonoscopy and corresponding and positive predictive values (PPVs).

Colonoscopy findings	Subjects *No.*	PPV %
Colorectal cancer	41	1.28
Advanced precancerous lesions (APLs)	75	2.34
Advanced neoplasia^a^	116	3.63
Nonadvanced adenomas	448	14.00
Polyps^b^	1586	49.56
Any neoplasia	2150	67.19
Enteritis^c^	195	6.09

a Advanced neoplasia was defined as cancer or APL.

b 691 confirmed by pathological diagnosis.

c 73 confirmed by pathological diagnosis.

### 3.3 Risk factors associated with colorectal neoplasia

Accumulated evidence has suggested that family/personal history and mucus or blood in stool are associated with increased risk of CRC (16, 17). To evaluate these risk factors in this screening population, we collected information through CRC risk assessment questionnaires collected from 98,668 subjects (see Methods for details).

**Figure 3:**
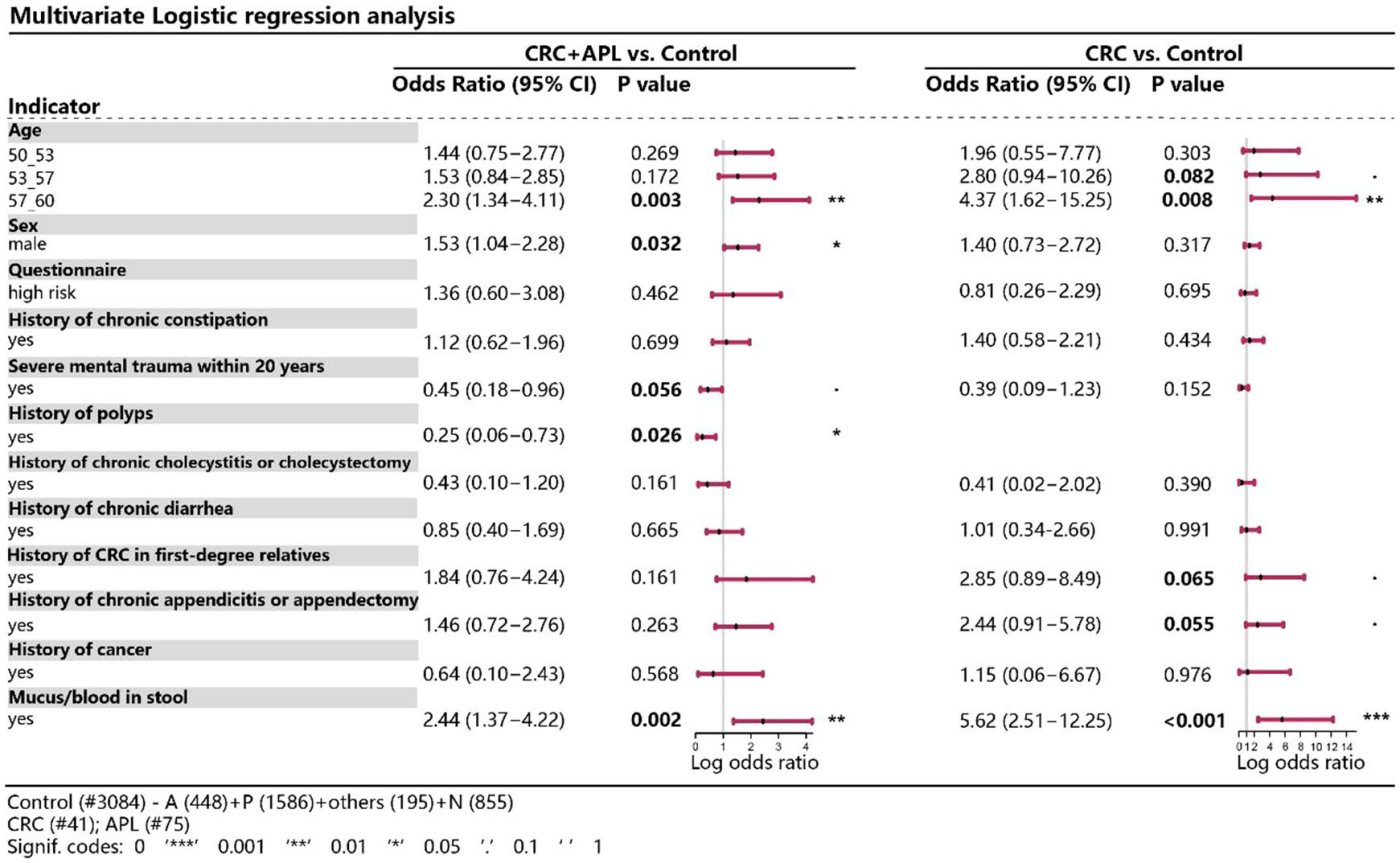
Multivariate logistic regression analysis of various variables between CRC/advanced neoplasia and control. *P < 0.05, **P < 0.01, ***P < 0.001.

We next attempted to identify potential risk factor of advanced colorectal neoplasia. As shown in **Figure 3**, older age, male gender, history of chronic constipation, family history of CRC in first-degree relatives, personal history of chronic appendicitis or appendectomy and personal history of bloody mucus in stools were positively associated with advanced colorectal neoplasia. Among them, we found that those who were older, male or had bloody mucus in stools also showed significantly higher risk of advanced colorectal neoplasia, showing odds ratio of 2.30 (95% CI 1.34-4.11), 1.53 (95% CI 1.04-2.28) and 2.44 (95% CI 1.37-4.22), respectively.

### 3.4 Methylation index in colorectal neoplasia

**Figure 4:**
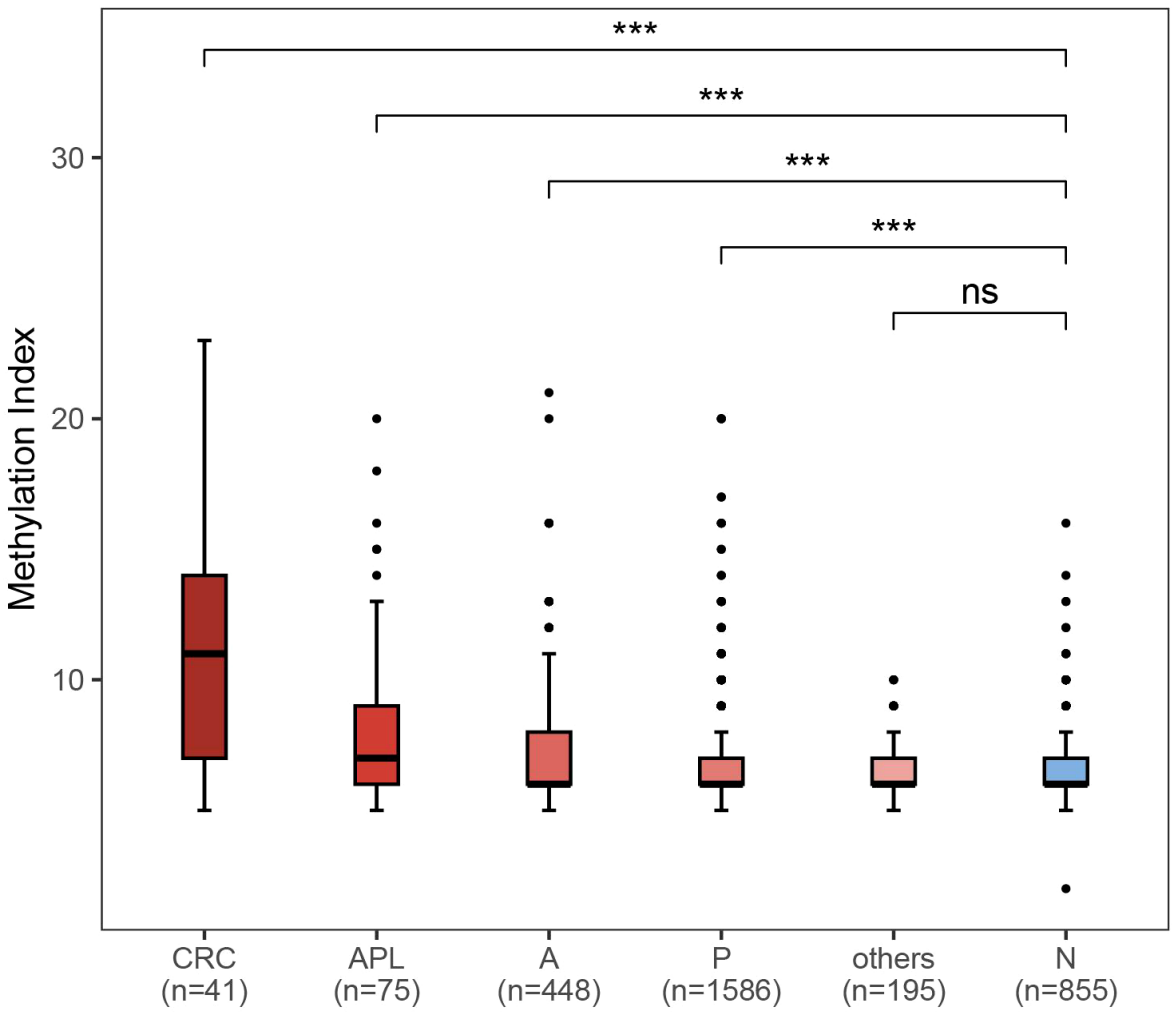
Comparison of methylation index between different colorectal neoplasm and healthy control. CRC: colorectal cancer (n = 41); APL: advanced precancerous lesions (n = 75); A: nonadvanced adenoma (n = 448); P: polyps (n = 1586); others: enteritis (n = 195); N: no findings in colonoscopy or pathological exam. ***P < 0.001; ns, not significant.

The result of fecal DNA test was determined by the methylation index score of three CRC-related genes, *SDC2*, *ADHFE1*, and *PPP2R5C* based on methylation status according to manufacturer’s protocol. A methylation index larger than 5 was considered as being at a higher risk of having CRC or APLs. To evaluate the association between methylation index and status of colorectal neoplasia, we compared the methylation indexes between participants with different disease status. In comparison to healthy individuals, the methylation indexes were considerably higher in subjects with neoplasia such as CRC (*P* < 0.001), APLs (*P* < 0.001), non-advanced adenoma (*P* < 0.001) and non-adenomatous polyp (*P* < 0.001), but not in those with enteritis (**Figure 4**).

## 4 Discussion

In this real-world study, we comprehensively analyzed the screening and diagnostic yields of a novel CRC screening strategy (fecal DNA test screening followed colonoscopy diagnosis) in an average-risk population. To the best of our knowledge, our study is the first to demonstrate the screening and diagnostic yield of such two-step strategy for CRC screening in a large-scale community-based program in China.

Colonoscopy is often considered the “gold standard” for CRC detection (6, 18). However, due to the low acceptance of colonoscopy by the general population, its availability and cost, it could be impractical to use colonoscopy as the standard-of-care screening approach in population-based CRC screening, especially in developing countries (19–21). The implementation of non-invasive screening tests in primary screening setting, such as FIT, questionnaires, and fecal DNA test, has emerging as alternative approach for large-scale population screening (22–24). However, effectiveness of such two-step screening strategy may be heavily compromised if the compliance rate of colonoscopy is low, after the primary screening tests return positive results. Previous report showed that non-compliance with colonoscopy after a positive fecal immunochemical test doubles the risk of dying from colorectal cancer (25). Notably, in our study, the participation rate of colonoscopy reached 71.93%, which was remarkably high compared to previous reports of population-based CRC screening studies in China: an earlier report of CRC screening program conducted in Zhejiang province between 2006-2008 reported a colonoscopy participation rate of 55.31% (26); more recent reports of CRC screening program utilizing FIT and/or risk assessment questionnaires as primary screening methods reported colonoscopy participation rates varying between 14.00% to 24.02% (6, 27–29), which were much lower than current study.

The high participation rate for colonoscopy in our study may be attributed to the following reasons: (1) Health publicity activities to promote CRC prevention in Wuhan city throughout this screening program, including public service text messages and science lectures to promote cancer screening and to improve the awareness of the target population; and (2) a convenient participation process of the screening program: the sample collection kit can be obtained from the neighborhood community health service center or street health center by making an appointment through mobile app. Those who screen positive will be advised to undergo colonoscopy diagnosis and will be given priority for making appointments for the procedure. (3) Non-invasive fecal DNA testing for CRC screening was adopted as the primary screening method. Stool testing has the advantages of being non-invasive, convenient, highly accurate, and no bowel preparation is needed. The results of our study suggest that increased public awareness, convenient participation procedure, and choice of non-invasive fecal DNA test as primary screening method can help improve participation rates for population-based CRC screening.

Importantly, only those aged between 45 to 60 years were included in current study, hence a lower diagnostic yield would be expected than we would see in an older population since incidence of adenoma and CRC significantly rises in population aged 60 and above (3, 30). Therefore, it is difficult to compare current study to previous reports of CRC screening cohorts in China using questionnaire or FIT-based strategy or colonoscopy alone (6, 31), which recruited participants aged between 40-69 years or 50-74 years. In future studies, it would be valuable to compare diagnostic yields of fecal DNA-based screening program to that of well-established screening strategies, using randomized controlled trial in the same population.

In terms of evidence-based quality indicators for colonoscopies, the adenoma detection rate (ADR) is currently regarded as one of the most important (32–34). In 2015, the recommended target ADR for all colonoscopies was increased to above 25% among people aged ≥ 50 years (34). Nevertheless, the above target rate is derived from data in western population, which has a high prevalence of CRC (10, 35). According to previous report, the prevalence of CRC in the United States is nearly twice of that in China, hence a lower ADR may be expected for Chinese population (36). Importantly, it was demonstrated that a reduced risk of CRC and death was associated with an increased ADR in CRC screening (37). The overall ADR in current study was 17.6% (564 precanceorus lesions was found out of 3200 colonoscopies). Although it was much lower than screening studies based on FIT conducted in Europe and North America (38), it was comparable to that of previous CRC screening programs conducted in China. For instance, an ADR of 11.49% was reported for a risk assessment questionnaire-based screening program (6), and an ADR of 31.4% was found for a FIT-based screening study (31). In future studies, it would be important to continue to monitor ADR to ensure the quality of colonoscopies.

In line with previous studies, we found that an older age, male gender, history of polyps, and history of mucous/blood in stool, were associated with the presence of CRC and/or APLs. For instance, older age has been well established as a risk factor for colorectal adenoma and CRC (6, 39, 40). Men were reported to be more likely to develop CRC than women, possibly due to a lack of the protection by estrogen or being more likely to have an unhealthy lifestyle such as smoking and alcohol consumption. In addition, androgens may be involved in the development of CRC by regulating the proliferation of intestinal epithelial cells (39–41). Song et al. found that individuals with a history of CRC precursors, such as polyps, had a 62% higher risk of developing CRC. We also found that history of polyps was associated with the incidence of CRC and APLs (*P* < 0.05).

Current study has some obvious limitations. First, our study was conducted in a single city and cannot reflect the regional diversity of the Chinese population, which may also limit the generalizability of our conclusion. Second, participation in screening program was voluntary, therefore selection bias cannot be ruled out. In addition, risk factor information was self-reported, and recall bias may be inevitable. Meanwhile, pathological diagnoses were lacking for some participants with polyps. It is generally recommended to submit all resected polyps for histopathological examination, since the polyp may have had a benign appearance at endoscopy but found to be invasive or advanced lesions after pathological examination (42). Therefore, missing of pathological examination for polyp patients may have caused underestimation of APL cases in current study. In the future, it would be desirable to further optimize the workflow to ensure all polyps be submitted for histopathological diagnosis. Another important limitation of current study is that no follow-up colonoscopies were conducted for those who screened negative with the fecal DNA test in this study, and therefore, we could not evaluate the sensitivity and specificity of the fecal test in this prospective cohort. In the future, such evaluation may be necessary for comprehensively evaluating the performance of Colotect^®^, and/or similar non-invasive fecal tests. Lastly, no head-to-head comparison with existing primary screening methods such as FIT or questionnaire-based system was conducted. Importantly, only those aged between 45 to 60 years were included in current study, hence a lower diagnostic yield would be expected than we would see in an older population since incidence of adenoma and CRC significantly rises in population aged 60 and above (3, 30). This made it difficult to compare the overall diagnostic yield of current study to previous reports of CRC screening cohorts in China using FIT or questionnaire-based strategy or colonoscopy alone (6, 27–29, 31), which recruited participants aged up to 69, 74, or 79 years old. Further studies are warranted to compare diagnostic yields and overall cost-effectiveness of fecal DNA-based screening approach to that of well-established screening strategies, using randomized controlled trial in the same population.

Despite these caveats, current study has several strengths. First, this is the first study to apply fecal DNA test in such a large-scale population-based screening program of CRC; screening procedures were all conducted under monitoring and strictly supervised by expert panel throughout the process, which ensured the reliability of the conclusion. Second, all researchers participating in the project received standardized training on reporting of clinical data, and standardized methods were adopted to collect questionnaires from participants, to ensure quality of data retrieved. Finally, collections of questionnaires allowed us to analyze association of known high risk factors with findings of diagnostic colonoscopy and revealed significant association with age, gender, history of polyps, and presence of mucus/blood in stool, which may provide insights into how future screening strategy could be optimized. For example, those with self-reported history of polyps and presence of mucus/blood in stool may be provided more supervision to encourage colonoscopy follow-up once they receive positive test results for fecal DNA assay.

In summary, we have applied non-invasive fecal DNA test as a primary screen method in a large-scale community-based population screening program of CRC. We showed that remarkably high participation rate of diagnostic colonoscopy was achieved following positive screening results of the fecal DNA test. We found that such strategy produced reasonable diagnostic yields, but further studies are needed to compare the novel strategy to existing CRC screening methods. We also showed that risk of CRC/APLs was associated with older age, male gender, history of polyps, and presence of mucus/blood in stool. Overall, our findings provided evidence for the feasibility of using fecal DNA test as a primary CRC screening tool and provided insights for designing and optimizing CRC screening strategies in the future.

## 5 Conclusions

This large-scale population-based CRC screening study using fecal DNA test as primary screening followed by diagnostic colonoscopy may help formulating effective CRC screening strategies in the future.

## Data availability statement

The original contributions presented in the study are included in the article/supplementary material. Further inquiries can be directed to the corresponding authors.

## Ethics statement

The studies involving humans were approved by Medical Ethics Committee of Wuhan Hospital of Integrated Traditional Chinese and Western Medicine. The studies were conducted in accordance with the local legislation and institutional requirements. The participants provided their written informed consent to participate in this study.

## Author contributions

SL: Conceptualization, Data curation, Investigation, Writing – review & editing. YFW: Data curation, Methodology, Writing – original draft, Writing – review & editing. YYW: Data curation, Methodology, Writing – review & editing, Software, Visualization. CD: Writing – original draft, Data curation, Methodology, Writing – review & editing. FL: Writing – original draft. HZ: Investigation, Writing – review & editing. XT: Investigation, Writing – review & editing. XD: Investigation, Writing – review & editing. MZ: Investigation, Writing – review & editing. DC: Investigation, Writing – review & editing. YL: Writing – review & editing, Investigation. RJ: Data curation, Methodology, Visualization, Writing – review & editing. DZ: Data curation, Methodology, Software, Visualization, Writing – review & editing. JP: Data curation, Methodology, Software, Visualization, Writing – review & editing. SZ: Data curation, Methodology, Software, Visualization, Writing – review & editing. LZ: Writing – review & editing. JW: Writing – review & editing. LW: Project administration, Supervision, Writing – review & editing. ZS: Conceptualization, Investigation, Project administration, Supervision, Writing – review & editing.
